# Supplementary material for: Development and Internal Validation of a Risk Prediction Model to Identify Myeloma Based on Routine Blood Tests: A Case-Control Study
Source: Cancers (Basel). 2023 Feb 3;15(3):975. doi: 10.3390/cancers15030975 (PMC9913376; doi:10.3390/cancers15030975)

## Supplementary tables and figures

**Table S1: Number of tests available up to 5-year prior to index date for myeloma patients, MGUS patients and controls**

| Blood test | Myeloma Number tests | Myeloma Number patients | Myeloma Median tests per patient | MGUS Number tests | MGUS Number patients | MGUS Median tests per patient | Controls Number tests | Controls Number patients | Controls Median tests per patient |
|---|---|---|---|---|---|---|---|---|---|
| Albumin | 4992 | 409 | 9 | 4493 | 392 | 8 | 11967 | 1418 | 5 |
| ALP | 4177 | 409 | 7 | 3958 | 392 | 7 | 11953 | 1418 | 5 |
| ALT | 3724 | 408 | 6 | 3621 | 391 | 6 | 11179 | 1413 | 4 |
| APTT | 1174 | 292 | 2 | 783 | 220 | 2 | 4497 | 958 | 2 |
| Basophil | 4887 | 408 | 8 | 4675 | 392 | 8 | 16399 | 1493 | 5 |
| Calcium | 2134 | 400 | 3 | 1928 | 382 | 3 | 5042 | 960 | 2 |
| Creatinine | 5655 | 411 | 9 | 5318 | 393 | 9 | 18489 | 1488 | 6 |
| CRP | 2304 | 365 | 3 | 2112 | 330 | 3 | 9120 | 1092 | 4 |
| D-Dimer | 89 | 71 | 1 | 63 | 46 | 1 | 239 | 161 | 1 |
| Derived Fibrinogen | 1083 | 291 | 2 | 737 | 218 | 2 | 4075 | 949 | 2 |
| eGFR | 5390 | 411 | 9 | 5004 | 393 | 9 | 17688 | 1487 | 6 |
| Eosinophil | 4928 | 409 | 8 | 4707 | 392 | 8 | 16469 | 1493 | 5 |
| ESR | 152 | 96 | 1 | 226 | 132 | 1 | 330 | 199 | 1 |
| Haemoglobin | 5038 | 409 | 8 | 4783 | 392 | 8 | 17600 | 1493 | 5 |
| Hypochromic | 4948 | 409 | 8 | 4712 | 392 | 8 | 16498 | 1493 | 5 |
| INR | 2215 | 301 | 3 | 2153 | 236 | 2 | 8475 | 986 | 3 |
| LDH | 280 | 186 | 1 | 311 | 219 | 1 | 172 | 90 | 1 |
| Lymphocyte | 4981 | 409 | 8 | 4741 | 392 | 8 | 16665 | 1493 | 5 |
| MCV | 4940 | 409 | 8 | 4698 | 391 | 8 | 16462 | 1493 | 5 |
| MCH | 4986 | 409 | 8 | 4730 | 391 | 8 | 16638 | 1493 | 5 |
| Monocyte | 4975 | 409 | 8 | 4739 | 392 | 8 | 16653 | 1493 | 5 |
| Neutrophil | 4984 | 409 | 8 | 4744 | 392 | 8 | 16683 | 1493 | 5 |
| PCV | 4957 | 409 | 8 | 4705 | 391 | 8 | 16521 | 1493 | 5 |
| Platelets | 4998 | 409 | 8 | 4742 | 392 | 8 | 16642 | 1493 | 5 |
| Prothrombin Time | 1091 | 291 | 2 | 755 | 218 | 2 | 4188 | 957 | 2 |
| PV | 539 | 275 | 2 | 655 | 274 | 2 | 754 | 402 | 1 |
| RDW | 4973 | 409 | 8 | 4723 | 391 | 8 | 16583 | 1493 | 5 |
| RBC | 4996 | 409 | 8 | 4734 | 391 | 8 | 16668 | 1493 | 5 |
| Urea | 5577 | 411 | 9 | 5303 | 393 | 9 | 18464 | 1488 | 6 |
| Urine Creatinine | 1027 | 329 | 2 | 1228 | 365 | 2 | 1327 | 423 | 2 |
| Vitamin D | 220 | 131 | 1 | 316 | 156 | 1 | 579 | 293 | 1 |
| WCC | 5008 | 409 | 8 | 4754 | 392 | 8 | 16717 | 1493 | 5 |

This table include all tests initially considered for this study

Abbreviations: ALP = alkaline phosphatase , ALT = alanine transaminase, APTT = activated partial thromboplastin time, CRP = C-reactive protein, eGFR = estimated glomerular filtration rate, ESR = erythrocyte sedimentation rate, INR = international normalised ratio, LDH = lactate dehydrogenase, MCV = mean cell volume, MCH = mean cell haemoglobin, PCV = packed cell volume, PV = plasma viscosity, RDW = red cell distribution width, RBC = red blood cell count, WCC = white cell count

**Table S2: Candidate predictors variables included in model development**

| Predictor variable | Units | Variable Type |
|---|---|---|
| Age at diagnosis/index date | Year | Continuous |
| Sex | | Factor (Male, Female) |
| Albumin | g/L | Continuous |
| ALP | iu/L | Continuous |
| ALT | iu/L | Continuous |
| Basophils | $10^9$/L | Continuous |
| Calcium | mmol/L | Continuous |
| Creatinine | Umol/L | Continuous |
| CRP | mg/L | Continuous |
| Eosinophils | $10^9$/L | Continuous |
| Haemoglobin | g/L | Continuous |
| Lymphocytes | $10^9$/L | Continuous |
| MCV | fL | Continuous |
| Monocytes | $10^9$/L | Continuous |
| Neutrophils | $10^9$/L | Continuous |
| Platelets | $10^9$/L | Continuous |
| WCC | $10^9$/L | Continuous |

**Figure S1: Flow chart of MGUS cohort**

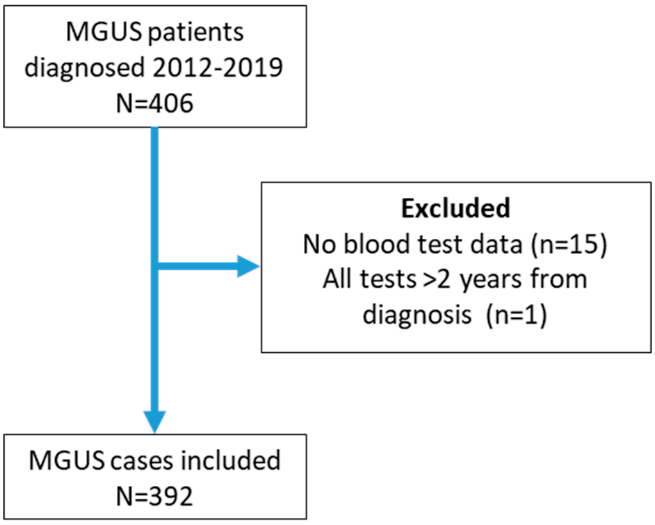

**Table S3: Baseline characteristics of MGUS cohort**

| Variable | MGUS cohort<br>N=392<br>Median (IQR) or n(%) | |
|---|---|---|
| **Age** | 75 | (66, 82) |
| **Sex** | | |
| Male | 224 | 57.1% |
| Female | 168 | 42.9% |
| **Ethnicity** | | |
| White British | 342 | 87.2% |
| Black | 19 | 4.9% |
| South Asian | 14 | 3.6% |
| Other and missing | 17 | 4.3% |
| **Total number of comorbidities** | | |
| 0 | 266 | 67.9% |
| 1 | 86 | 21.9% |
| 2 | 34 | 8.7% |
| 3+ | 6 | 1.5% |
| **Blood tests** | | |
| Albumin | 40.0 | (37.0, 42.0) |
| ALP | 121.5 | (82.0, 202.0) |
| ALT | 19.0 | (14.0, 26.0) |
| Basophils | 0.04 | (0.03, 0.05) |
| Calcium | 2.39 | (2.31, 2.45) |
| Creatinine | 80.0 | (63.0, 100.3) |
| CRP | 5.00 | (5.0, 11.58) |
| Eosinophils | 0.13 | (0.08, 0.22) |
| Haemoglobin | 131.0 | (118.0, 141.5) |
| Lymphocytes | 1.51 | (1.12, 1.96) |
| MCV | 94 | (90, 98) |
| Monocytes | 0.38 | (0.30, 0.50) |
| Neutrophils | 4.16 | (3.25, 5.40) |
| Platelets | 242.0 | (189.5, 300.0) |
| WCC | 6.55 | (5.42, 8.14) |

**Figure S2: Trend plots for blood test for cases and controls, mean values (and 95% CIs) within 90 day periods**

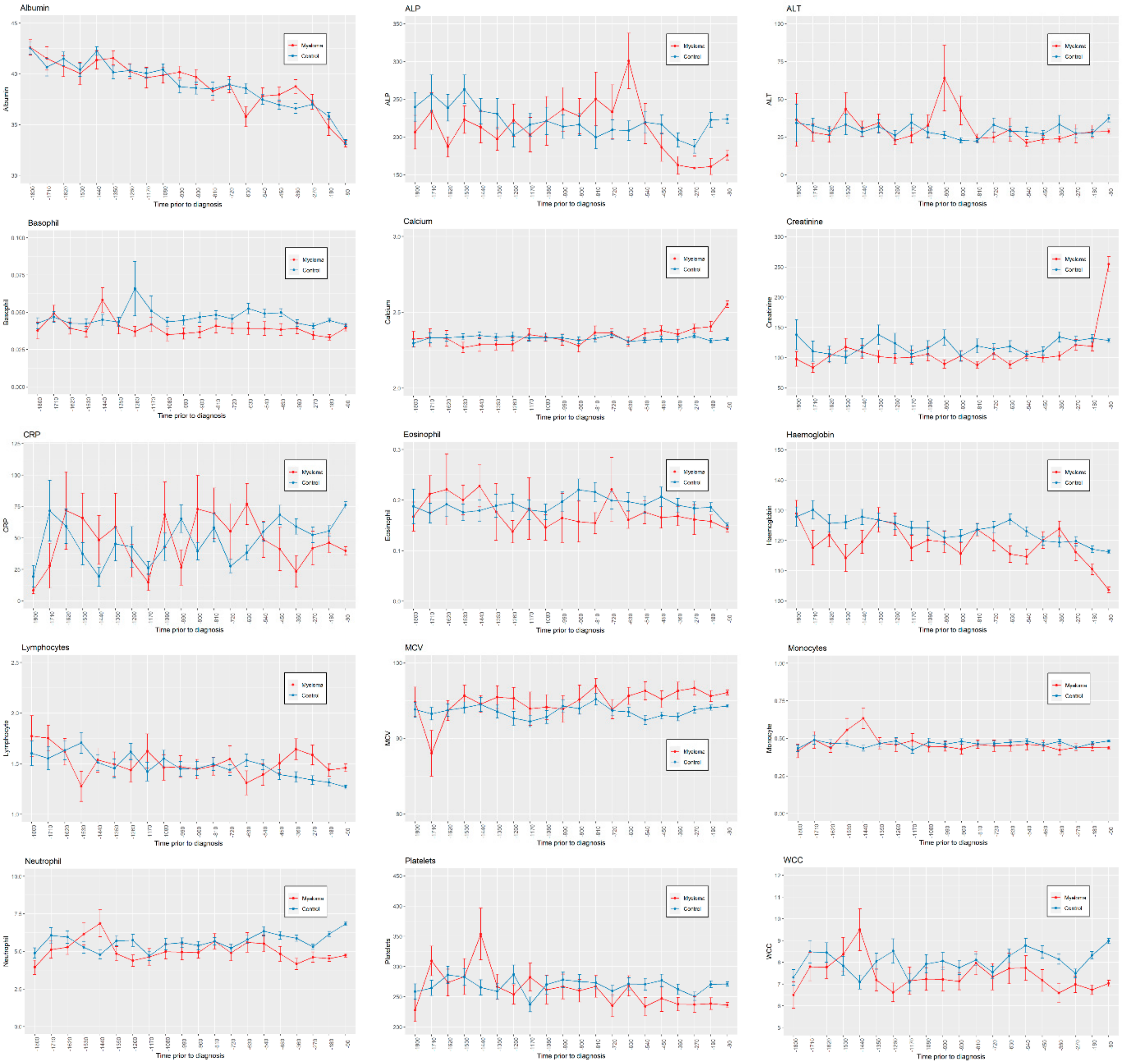

## Figure S3: Box plot of distribution of blood tests for cases and controls for test results included in the model

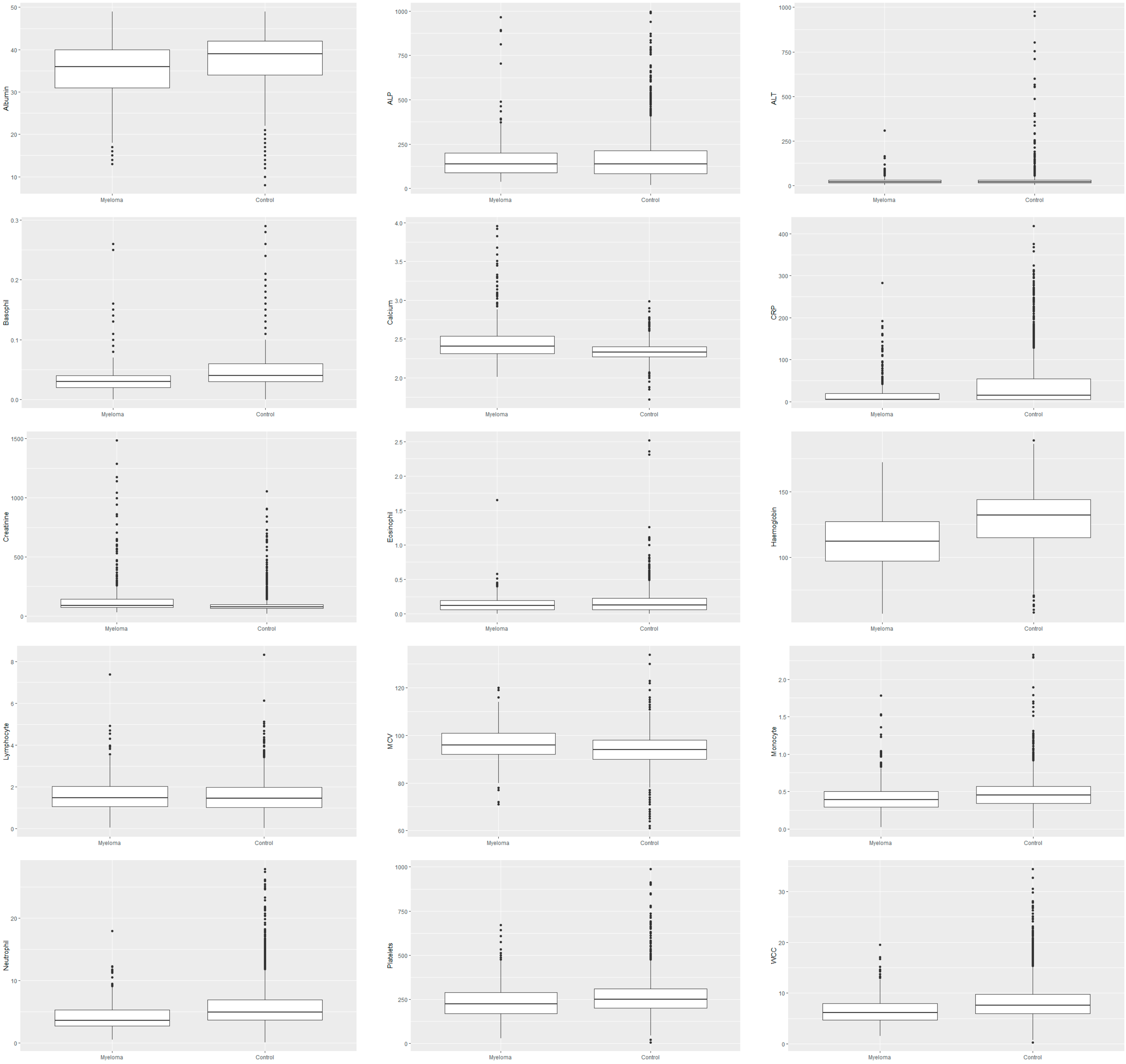

**Table S4: Most frequent requesting departments for blood test results used in model development**

| Cases | | Controls | |
|---|---|---|---|
| **Requesting department** | **% of tests** | **Requesting department** | **% of tests** |
| Primary Care | 36% | Primary Care | 34% |
| Medicine | 19% | Medicine | 15% |
| Haematology Outpatients | 9% | Accident and emergency | 9% |
| Elderly care | 7% | Cardiology | 8% |
| Renal | 6% | Elderly care | 4% |
| Respiratory | 5% | Respiratory | 4% |
| Accident and emergency | 5% | Spinal surgery | 4% |
| Haematology inpatients | 3% | General surgery | 3% |
| Orthopaedics | 2% | Orthopaedics | 3% |
| General surgery | 2% | Renal | 2% |

Each component of the FBC is counted as a separate test

**Table S5: Number and percentage with missing data for blood tests in cases and controls.**

| | Overall | | Cases | | Controls | |
|---|---|---|---|---|---|---|
| **Test** | **n** | **%** | **n** | **%** | **n** | **%** |
| Albumin | 98 | 5.3 | 8 | 2.2 | 90 | 6.0 |
| ALP | 98 | 5.3 | 8 | 2.2 | 90 | 6.0 |
| ALT | 110 | 5.9 | 15 | 4.1 | 95 | 6.4 |
| Basophil | 18 | 1.0 | 12 | 3.3 | 6 | 0.4 |
| Calcium | 656 | 35.4 | 60 | 16.3 | 596 | 40.0 |
| Creatinine | 11 | 0.6 | 3 | 0.8 | 8 | 0.5 |
| CRP | 510 | 27.5 | 89 | 24.3 | 421 | 28.3 |
| Eosinophil | 16 | 0.9 | 11 | 3.0 | 5 | 0.3 |
| Haemoglobin | 15 | 0.8 | 11 | 3.0 | 4 | 0.3 |
| Lymphocyte | 15 | 0.8 | 11 | 3.0 | 4 | 0.3 |
| MCV | 16 | 0.9 | 12 | 3.3 | 4 | 0.3 |
| Monocyte | 15 | 0.8 | 11 | 3.0 | 4 | 0.3 |
| Neutrophil | 15 | 0.8 | 11 | 3.0 | 4 | 0.3 |
| Platelets | 15 | 0.8 | 11 | 3.0 | 4 | 0.3 |
| WCC | 15 | 0.8 | 11 | 3.0 | 4 | 0.3 |

**Table S6: Model coefficients for final model based**

| Variable | Coefficient (log(OR)) | Standard error |
|---|---|---|
| Intercept | -10.617 | 4.401 |
| Sex | -0.255 | 0.177 |
| Age | 0.006 | 0.014 |
| Age spline 1 | -0.017 | 0.016 |
| Albumin | -0.019 | 0.023 |
| Albumin spline 1 | -0.006 | 0.026 |
| ALP | 0.007 | 0.002 |
| ALP spine 1 | -0.009 | 0.003 |
| ALT | 0.014 | 0.015 |
| ALT spline 1 | -0.023 | 0.021 |
| Basophil | -27.403 | 8.695 |
| Basophil spline 1 | 21.675 | 11.557 |
| Calcium | 1.922 | 1.549 |
| Calcium spline 1 | 3.372 | 1.744 |
| Creatinine | 0.018 | 0.006 |
| Creatinine spline 1 | -0.024 | 0.009 |
| CRP | -0.029 | 0.008 |
| CRP spline 1 | 0.050 | 0.022 |
| Eosinophil | -0.841 | 2.204 |
| Eosinophil spline 1 | -0.733 | 2.700 |
| Haemoglobin | -0.023 | 0.008 |
| Haemoglobin spline 1 | -0.029 | 0.010 |
| Lymphocytes | 1.740 | 0.420 |
| Lymphocytes spline 1 | -1.109 | 0.377 |
| MCV | 0.071 | 0.025 |
| MCV spline 1 | -0.058 | 0.030 |
| Monocytes | 1.185 | 1.259 |
| Monocytes spline 1 | 0.747 | 1.446 |
| Neutrophil | 0.150 | 0.282 |
| Neutrophil spline 1 | -0.271 | 0.289 |
| Platelets | 0.003 | 0.002 |
| Platelets spine 1 | -0.003 | 0.002 |
| WCC | -0.535 | 0.271 |
| WCC spline 1 | 0.457 | 0.235 |

Restricted cubic spline knot positions:
Age: 52, 70, 84, Albumin: 28, 38, 44, ALP: 64,137, 302, ALT: 12, 21, 48, Basophil: 0.02, 0.04, 0.08, Calcium: 2.21, 2.34, 2.51, Creatinine: 54, 78, 170, CRP: 5.1, 30, 155, Eosinophil, 0.02, 0.13, 0.33, Haemoglobin: 96, 129, 153, Lymphocytes: 0.74, 1.48, 2.63, MCV: 87, 94, 103, Monocytes: 0.26, 0.43, 0.73, Neutrophil: 2.55, 4.75, 9.52, Platelets: 152, 244, 377, WCC: 4.53, 7.32, 12.35

**Figure S4: Predicted probabilities cases and controls (over imputed models)**

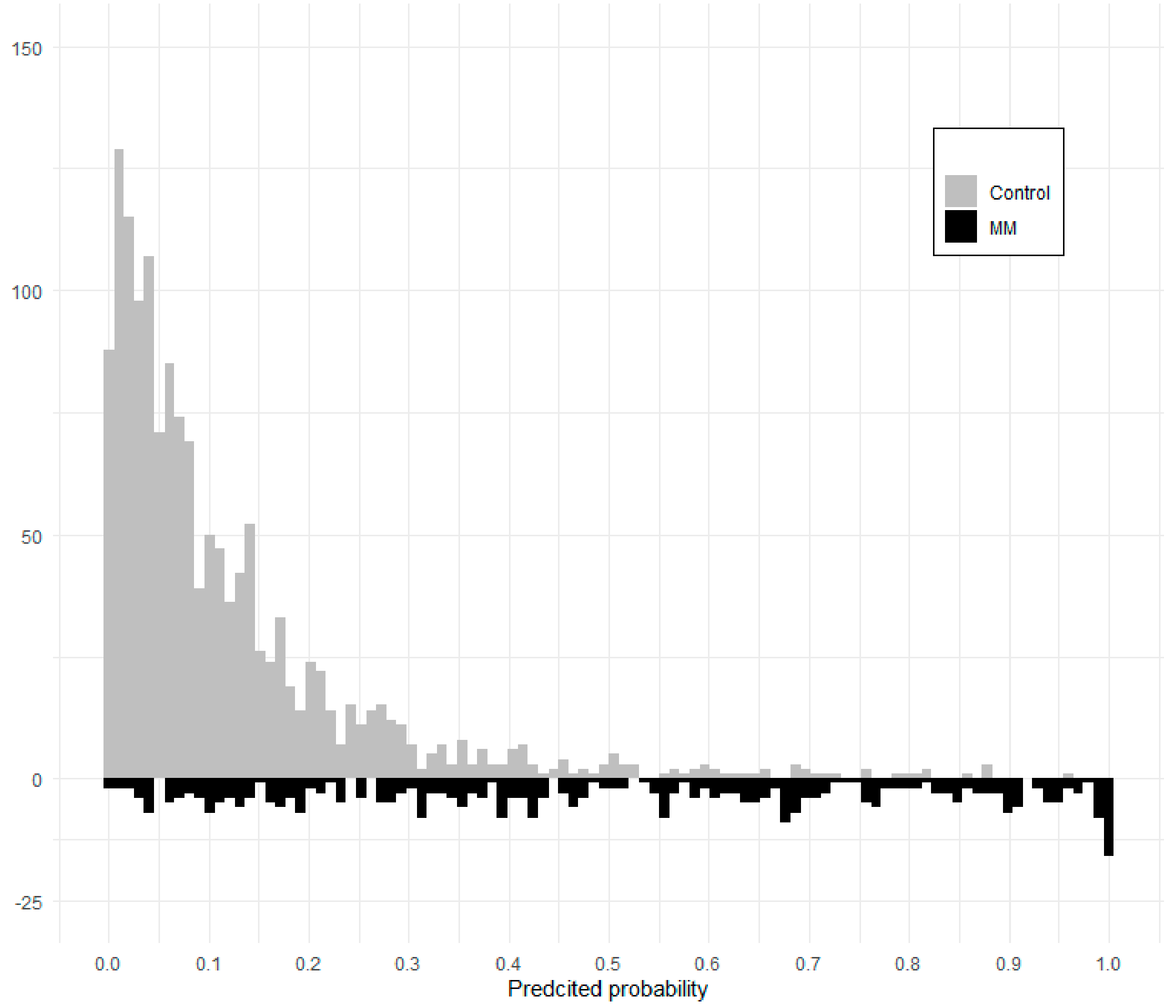

**Table S7: Estimated prevalence based on published studies and national cancer data**

| Author, year | Study population | Estimated prevalence/incidence |
|---|---|---|
| Koshiaris et al, 2021 (1) | CRPD >40 years, registered GP for >1 year, Two FBCs within 1 year between 2000 and 2014 with 2 years follow-up | 737 cases Total population 1,281,926 57 cases per 100,000 (paper used 60 cases per 100,00 pop for diagnostic performance statistics) |
| Seesaghur et al, 2021 (2) | CRPD, aged 18+, registered GP for >2 years, Newly diagnosed MM between 2006 and 2016 | 2646 cases Total population 17,756,119 15 cases per 100,000 |
| NHS Digital, 2022 (3) | England cancer registrations 2019 All ages | Age standardised incidence rate Males 12.9 per 100,000 Females 8.2 per 100,000 |

**References**


1. Koshiaris C, Van den Bruel A, Nicholson BD, Lay-Flurrie S, Hobbs FR, Oke JL. Clinical prediction tools to identify patients at highest risk of myeloma in primary care: a retrospective open cohort study. Br J Gen Pract. 2021;71(706):e347-e55.
2. Seesaghur A, Petruski-Ivleva N, Banks VL, Wang JR, Abbasi A, Neasham D, et al. Clinical features and diagnosis of multiple myeloma: a population-based cohort study in primary care. BMJ Open. 2021;11(10):e052759.
3. National Disease Registration Service NHS Digital. CancerData. Cancer Incidence 2019. https://www.cancerdata.nhs.uk/incidence_and_mortality 2022

**Table S8: Total number to additionally test at different prevalence estimates and probability thresholds for each model**

| | | Prevalence per 100,000 | | |
|---|---|---|---|---|
| **Model** | **Threshold** | **10** | **15** | **60** |
| **Full model** | 0.1 | 39805 | 39808 | 39830 |
| | 0.2 | 17706 | 17709 | 17736 |
| | 0.4 | 5205 | 5208 | 5232 |
| **Calcium removed** | 0.1 | 44404 | 44407 | 44427 |
| | 0.2 | 21506 | 21508 | 21534 |
| | 0.4 | 5805 | 5807 | 5830 |
| **Over 60s only** | 0.1 | 38405 | 38408 | 38432 |
| | 0.2 | 19406 | 19409 | 19337 |
| | 0.4 | 5706 | 5709 | 5736 |
| **MGUS as controls** | 0.1 | 36105 | 36107 | 36129 |
| | 0.2 | 14006 | 14009 | 14136 |
| | 0.4 | 4004 | 4006 | 4026 |
| **MGUS as controls and calcium removed** | 0.1 | 40604 | 40607 | 40627 |
| | 0.2 | 17205 | 17208 | 17233 |
| | 0.4 | 4304 | 4306 | 4324 |
| **Over 60s only, MGUS as controls, all predictors** | 0.1 | 36005 | 36008 | 36031 |
| | 0.2 | 15406 | 15409 | 15436 |
| | 0.4 | 5104 | 5107 | 5126 |
| **Over 60s only, MGUS as controls, calcium removed** | 0.1 | 39305 | 39307 | 39329 |
| | 0.2 | 18506 | 18508 | 18634 |
| | 0.4 | 5404 | 5406 | 5426 |

**Figure S5: Number needed to additionally test to diagnose 1 case at different prevalence estimates and probability thresholds for each model**

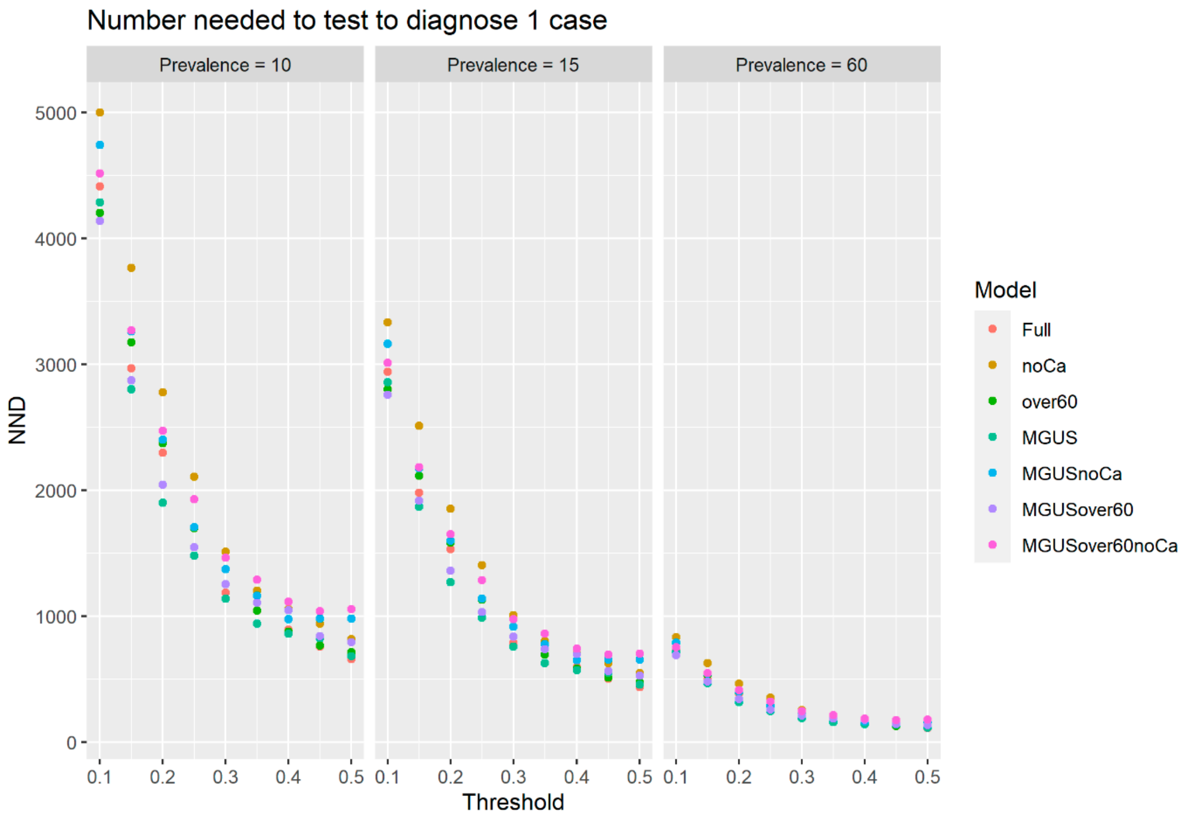

Supplement: Supplementary file 1 [file cancers-15-00975-s001.zip › cancers-2059473-supplementary.pdf]
